# Synergistic Antifungal Properties, Chemical Composition, and Frontier Molecular Orbital Analysis of Essential Oils from Lemongrass, Kaffir Lime, Lime, Dill, and Shatavari Against *Malassezia furfur*

**DOI:** 10.3390/ijms26125601

**Published:** 2025-06-11

**Authors:** Sarin Tadtong, Rada Chantavacharakorn, Sarocha Khayankan, Puriputt Akachaipaibul, Wanna Eiamart, Weerasak Samee

**Affiliations:** 1 Department of Pharmacognosy, Faculty of Pharmacy, Srinakharinwirot University, Nakhonnayok 26120, Thailand; sarin@g.swu.ac.th (S.T.); puriputt.akachaipaibul@g.swu.ac.th (P.A.); 2 Department of Pharmaceutical Chemistry, Faculty of Pharmacy, Srinakharinwirot University, Nakhonnayok 26120, Thailand; rada.da125@gmail.com (R.C.); sarocha_khayankan@hotmail.com (S.K.); 3 Chula Pharmacokinetic Research Center, Faculty of Medicine, Chulalongkorn University, Bangkok 10330, Thailand; wanna.e@chula.ac.th

**Keywords:** essential oil, synergism, anti-fungal, gas chromatography, mass spectrometry, dandruff, citral, citronellal, molecular orbital, electrophile

## Abstract

This study explores the chemical composition and synergistic anti-fungal properties of essential oils from the aerial parts of Satavari (*Asparagus racemosus* Willd.), Dill (*Anethum graveolens* L.), and lemongrass (*Cymbopogon citratus* Stapf), along with the peels of Lime (*Citrus aurantifolia* (Christm.)) and Kaffir lime (*Citrus hystrix* DC), as well as the leaves of *Citrus hystrix* DC, against *Malassezia furfur*, a yeast linked to dandruff and seborrheic dermatitis. Gas chromatography-mass spectrometry (GC-MS) identified key volatile compounds within these oils. In vitro anti-fungal assays evaluated their efficacy individually and in combinations using checkerboard dilution techniques to assess synergy. Results indicated significant antifungal activity, with lemongrass exhibiting the strongest effect (MIC of 0.125% *v*/*v*). Notably, a 1:1 combination of lemongrass and kaffir lime essential oils showed synergism, reducing the MIC to 0.0625% *v*/*v*. The antifungal activity was primarily attributed to citral and citronellal, with MICs of 0.03125% *v*/*v* and 0.125% *v*/*v*, respectively. Molecular orbital analysis revealed that the higher energy levels of the lowest unoccupied molecular orbitals (LUMOs) in citral correlate with greater antifungal efficacy, likely due to its enhanced electrophilicity, facilitating nucleophilic interactions with *M. furfur*’s cellular components. These findings highlight potential applications of essential oil combinations in antifungal therapies.

## 1. Introduction

The genus *Malassezia* comprises lipophilic yeasts that require lipids for growth, predominantly affecting regions such as the upper chest, back, arms, and neck. The proliferation of *Malassezia* is influenced by factors including elevated temperatures and increased perspiration—a condition known as hyperhidrosis—which can induce morphological changes from spherical to filamentous forms. When infecting scalp hair follicles, *Malassezia* can lead to dandruff and scalp inflammation [1]. The development of anti-dandruff shampoos utilizing essential oils has gained prominence due to a growing consumer preference for natural over synthetic ingredients. Various essential oils, such as tea tree and clove oils, exhibit potent antifungal activities against *Malassezia furfur*, the primary pathogen associated with dandruff [2,3,4]. Patchouli essential oil has also shown promise owing to its antimicrobial properties. Additionally, oils like peppermint contribute to scalp health through anti-inflammatory and circulation-enhancing effects [5,6]. Clinical evidence highlights lemongrass oil’s capacity to reduce scalp flakiness, albeit less effectively than tea tree oil [4]. Synergistic combinations of these essential oils can enhance anti-fungal efficacy, improving formulations of anti-dandruff products [7]. Incorporating other natural ingredients, such as coconut oil—known for promoting beneficial scalp microbiota—may further optimize formulations [8,9]. The chemical complexity of essential oils, comprising phenolics, terpenes, and other compounds, underpins their antifungal and biological activities [10,11,12,13,14]. Notably, agents like thymol and carvacrol exhibit fungicidal effects, making them suitable for therapeutic applications [15]. Beyond anti-fungal activity, essential oils possess antioxidants and anti-inflammatory properties that support skin health and mitigate infection-associated inflammation [12,16].

Several types of home garden vegetables contain essential oils that are easily accessible, abundant, and cost-effective. Numerous studies have reported the antifungal properties of these vegetables. For instance, dill (*Anethum graveolens* L.) essential oil demonstrates potent antifungal activity against *Aspergillus flavus*, with exposure causing significant cellular alterations, including damage to cell walls, plasma membranes, and organelles [17]. Additionally, extracts from *Asparagus racemosus* have shown antifungal effects against dermatophytes such as *Microsporum gypseum* and *Trichophyton mentagrophytes* [18]. Lime (*Citrus aurantifolia*) peels’ essential oil exhibits notable antifungal activity against *Aspergillus flavus* and *Penicillium chrysogenum* [19]. Rahayu et al. (2024) specifically investigated the antifungal properties of *Citrus hystrix* peel extract against *M. furfur*, demonstrating promising results that suggest its potential as a natural therapeutic agent for skin infections caused by this pathogen [20]. Furthermore, Rhimi et al. (2022) evaluated the antifungal activity of *Cymbopogon citratus* (lemongrass) essential oil against various fungi, including *M. furfur*. Their findings indicated that the essential oil, particularly its component citral, plays a significant role in inhibiting fungal growth [21]. Understanding the chemical composition and synergistic interactions of these essential oils is crucial for developing effective treatments for dermatological conditions associated with *Malassezia* spp. Studying their compositions and synergistic interactions is essential for advancing effective treatments for *Malassezia*-related dermatological conditions.

The utilization of frontier molecular orbital (FMO) analysis is essential for understanding the interactions between molecular parameters and the antifungal activities of various active compounds. FMOs, specifically the highest occupied molecular orbital (HOMO) and the lowest unoccupied molecular orbital (LUMO), provide significant insights into the electronic properties of compounds and their biological activities. The energy gap between HOMO and LUMO is a critical determinant of the reactivity and stability of compounds [22,23]. Moreover, the position and distribution of electrons within these orbitals are associated with a compound’s interaction with biological targets, such as vital fungal enzymes [24,25]. From an FMO perspective, variations in electronic parameters directly affect antifungal efficacy [26]. FMO analysis thus serves as a crucial tool in drug discovery, offering valuable insights into the antifungal activity of compounds based on their electronic structure. This relationship underscores the potential for targeted modifications to optimize the efficacy of antifungal agents through FMO analysis.

This study aimed to identify novel combinations of essential oils for anti-dandruff applications by examining their chemical compositions and inhibitory activities against *M. furfur*. The essential oils, obtained through hydro-distillation from the aerial parts of Shatavari, dill, and lemongrass, as well as the peels of lime and Kaffir lime, along with the leaves of Kaffir lime, were analyzed. Additionally, the research investigated the synergistic effects among these oils and explored the relationship between their chemical compositions and inhibitory activities against *M. furfur*. This study also examined the interactions between frontier molecular orbital parameters and the antifungal activities of the active compounds.

## 2. Results

### 2.1. Extraction Yields of Essential Oils

The extraction of essential oils from lemongrass leaves, kaffir lime peels, kaffir lime leaves, lime peels, dill aerial parts, and Shatavari aerial parts using the hydro-distillation method produced variable yields. The essential oil obtained from dill aerial parts exhibited the highest yield at 1.08% *w*/*w*, followed by kaffir lime peels at 0.86% *w*/*w*, lime peels at 0.68% *w*/*w*, Shatavari aerial parts at 0.65% *w*/*w*, lemongrass at 0.61% *w*/*w*, and kaffir lime leaves at 0.27% *w*/*w*. These findings are summarized in Table 1.

### 2.2. Chemical Composition of Essential Oils

**Figure 1:**
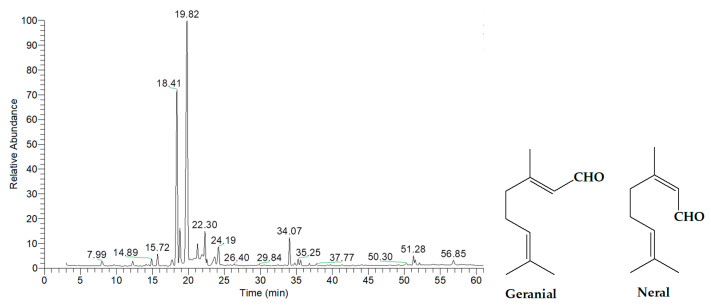
GC-MS chromatogram of essential oil extracted from lemongrass aerial parts.

**Figure 2:**
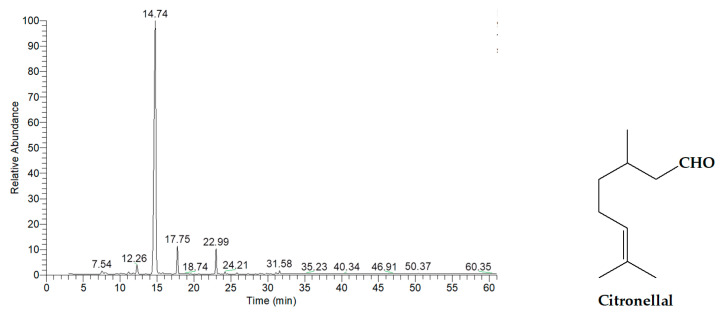
GC-MS chromatogram of essential oil extracted from kaffir lime leaves.

**Figure 3:**
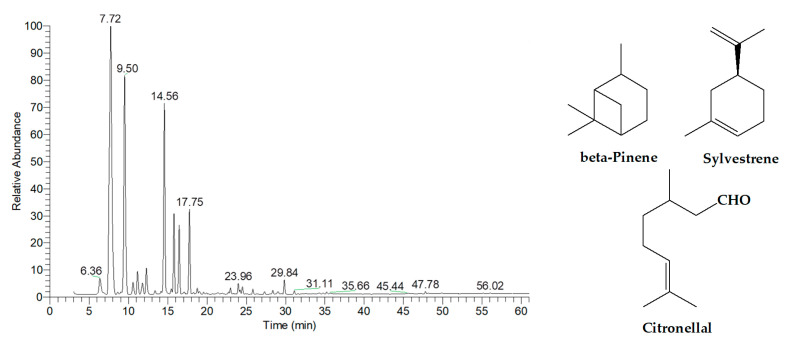
GC-MS chromatogram of essential oil extracted from kaffir lime peels.

**Figure 4:**
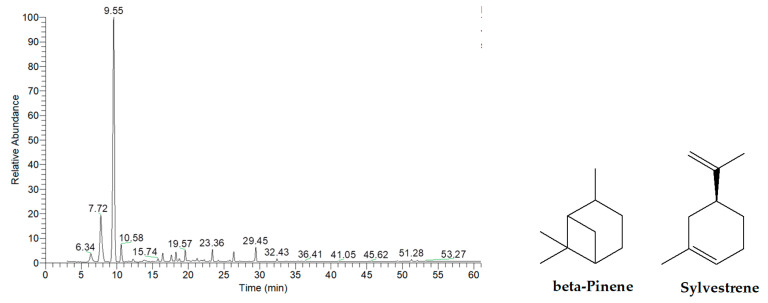
GC-MS chromatogram of essential oil extracted from lime peels.

**Figure 5:**
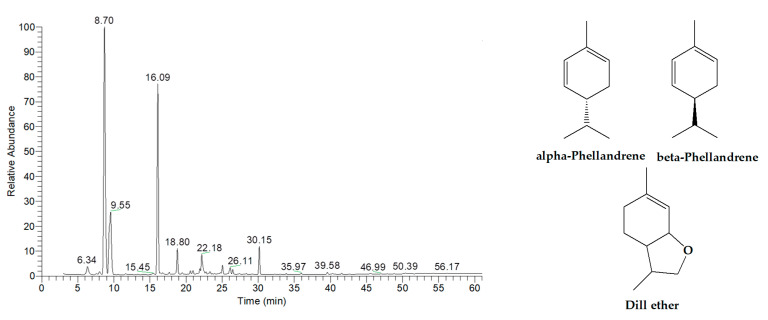
GC-MS chromatogram of essential oil extracted from dill aerial parts.

**Figure 6:**
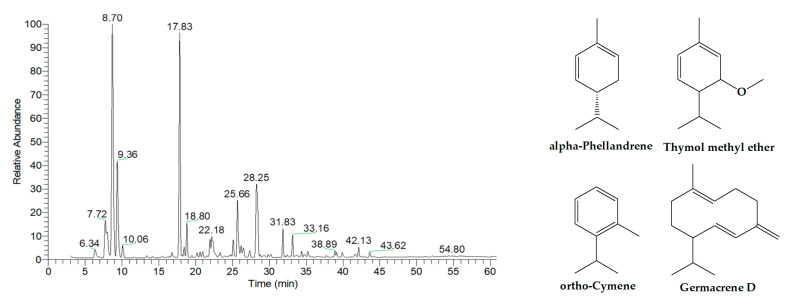
GC-MS chromatogram of essential oil extracted from Shatavari aerial parts.

The chemical composition of the essential oils was extensively analyzed using gas chromatography-mass spectrometry (GC-MS), and the results are summarized in Table 2, Table 3, Table 4, Table 5, Table 6 and Table 7, with corresponding GC-MS chromatograms shown in Figure 1, Figure 2, Figure 3, Figure 4, Figure 5 and Figure 6. As shown in Figure 1 and Table 2, the essential oil extracted from lemongrass aerial parts consisted predominantly of oxygenated monoterpenes, accounting for 92.41% of the total composition. The two major components were geranial, which constituted 45.03% of the oil and was detected at a retention time (RT) of 19.82 min, and neral, which comprised 27.07% and was detected at 18.41 min. These two compounds, optical isomers of each other, are collectively known as citral.

As shown in Figure 2 and Table 3, the essential oil obtained from kaffir lime leaves consisted predominantly of oxygenated monoterpenes, accounting for 97.62% of the total composition. The major component was citronellal, which constituted 83.76% of the oil and was detected at a retention time of 14.74 min.

As depicted in Figure 3 and Table 4, the essential oil extracted from kaffir lime peels consisted predominantly of monoterpene hydrocarbons and oxygenated monoterpenes, accounting for 60.62% and 35.94% of the total composition, respectively. The three major components identified were beta-pinene (29.49%, RT = 7.72 min), sylvestrene (20.77%, RT = 9.50 min), and citronellal (14.02%, RT = 14.56 min).

As illustrated in Figure 4 and Table 5, the essential oil extracted from lime peels consisted predominantly of monoterpene hydrocarbons and oxygenated monoterpenes, accounting for 83.92% and 11.45% of the total composition, respectively. The two major components identified were sylvestrene (62.29%, RT = 9.55 min) and beta-pinene (15.33%, RT = 7.72 min).

As shown in Figure 5 and Table 6, the essential oil extracted from dill aerial parts consisted predominantly of monoterpene hydrocarbons and oxygenated monoterpenes, accounting for 65.06% and 27.11% of the total composition, respectively. The three major components identified were alpha-phellandrene (43.54%, RT = 8.70 min), dill ether (25.24%, RT = 16.09 min), and beta-phellandrene (10.46%, RT = 9.55 min).

As presented in Figure 6 and Table 7, the essential oil extracted from Shatavari aerial parts consisted predominantly of monoterpene hydrocarbons and oxygenated monoterpenes, accounting for 45.55% and 29.10% of the total composition, respectively. The four major components identified were alpha-phellandrene (26.14%, RT = 8.70 min), thymol methyl ether (18.07%, RT = 17.82 min), ortho-cymene (10.30%, RT = 9.36 min), and germacrene D (10.03%, RT = 28.33 min).

For a comparative analysis of chemical classes across essential oil samples (Table 2, Table 3, Table 4, Table 5, Table 6 and Table 7), oxygenated monoterpenes were found to be the dominant class in lemongrass aerial parts and kaffir lime leaves. Conversely, monoterpene hydrocarbons were predominant in the essential oils from kaffir lime peels, lime peels, dill aerial parts, and Shatavari aerial parts, with oxygenated monoterpenes identified as the second most abundant class in these oils.

### 2.3. The Inhibitory Activity of Essential Oils Against Malassezia furfur

As detailed in Table 8, the essential oils from lemongrass, kaffir lime leaves, kaffir lime peels, lime peels, and dill aerial parts demonstrated substantial anti-fungal activity against *M. furfur*, with no microbial growth observed (NMGO). Conversely, the essential oil obtained from Shatavari aerial parts exhibited significantly lower efficacy, with an inhibition zone diameter of 1.05 ± 0.12 cm. Subsequently, the six essential oils were evaluated for their inhibitory effects on *M. furfur* through the determination of their minimum inhibitory concentrations (MICs) using the broth microdilution method. Based on these MIC values, as summarized in Table 8, the most potent essential oil was derived from lemongrass, exhibiting an MIC of 0.125% *v*/*v*.

### 2.4. Synergistic Effects of Combined Essential Oils on Malassezia furfur

According to Table 9, the antifungal activity, assessed through MIC and the Sum of Fractional Inhibitory Concentrations (ΣFIC) values of the combined essential oils against *M. furfur*, revealed that the most effective formulations were the mixtures of lemongrass oil with kaffir lime leaf essential oil, lemongrass essential oil with dill essential oil, and lemongrass essential oil with Shatavari essential oil, all in a 1:1 ratio. These combinations exhibited identical MIC values of 0.0625% *v*/*v*. The calculated ΣFIC for these combinations was 0.75, which was less than 1, indicating a synergistic interaction among the essential oils. This suggested that the combined formulations in a 1:1 ratio enhanced their antifungal effects against *M. furfur*.

**Figure 7:**
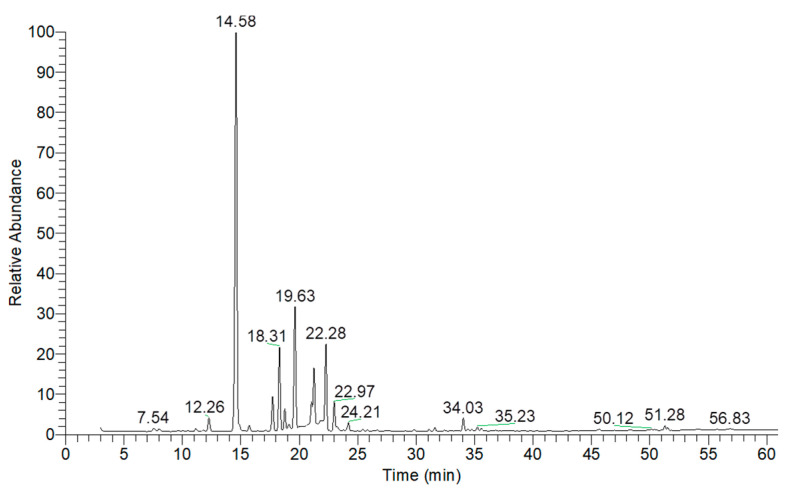
GC-MS chromatogram of combined lemongrass and kaffir lime leaf essential oils in a 1:1 ratio.

The three essential oil formulations that demonstrated synergistic effects and exhibited an MIC of 0.0625% were analyzed for their constituents. One such formulation consisted of a mixture of lemongrass and kaffir lime leaf essential oils in a 1:1 ratio. The primary components identified within this mixture included the top three constituents: citronellal (49.72%), geranial (12.68%), and neral (8.33%) (see Figure 7).

**Figure 8:**
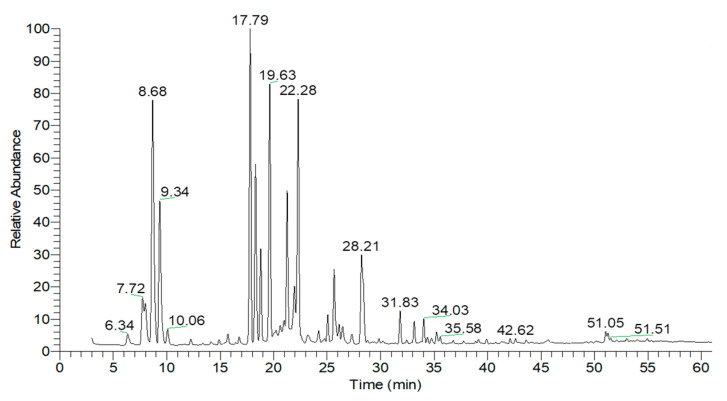
GC-MS chromatogram of combined lemongrass and Shatavari essential oils in a 1:1 ratio.

The combined essential oils of lemongrass and Shatavari in a 1:1 ratio were analyzed to identify their predominant constituents. The three major components included alpha-phellandrene (14.65%), thymol methyl ester (13.40%), and geranial (11.23%) (see Figure 8).

**Figure 9:**
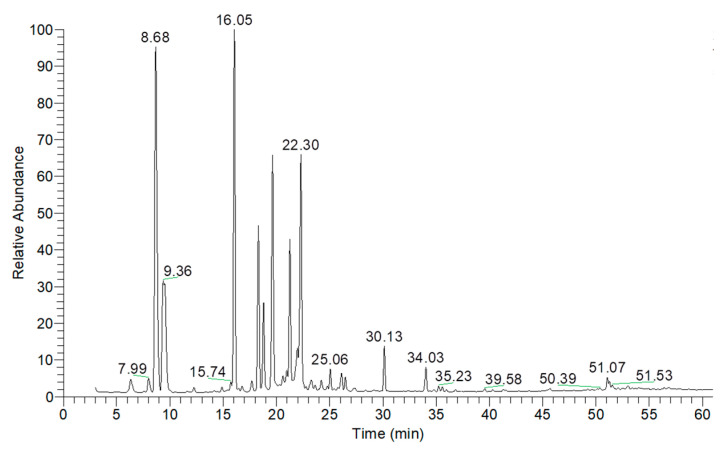
GC-MS chromatogram of combined lemongrass and dill essential oils in a 1:1 ratio.

The combined essential oils of lemongrass and dill in a 1:1 ratio were analyzed to determine their primary constituents. The three major components identified include alpha-phellandrene (21.67%), dill ether (15.83%), and geranial (11.09%) (see Figure 9).

The essential oil combinations of lemongrass with Shatavari and lemongrass with dill exhibited undesirable odors; hence, the mixture of lemongrass and kaffir lime leaf essential oils in a 1:1 ratio was selected for further investigation. Antimicrobial assays conducted against *M. furfur* identified citral—comprising geranial (trans-citral) and neral (cis-citral)—and citronellal as the primary active constituents in this essential oil mixture. As presented in Table 5, the MIC of citral was determined to be 0.03125% *v*/*v*, whereas citronellal exhibited an MIC of 0.125% *v*/*v*. Further evaluation of their combined effects revealed synergistic interactions in mixtures with ratios of 1:3 and 3:1, achieving MIC values of 0.0625% *v*/*v* and 0.03125% *v*/*v*, respectively. These findings indicated that the conjugated aldehyde structure in citral was more effective in inhibiting *M. furfur* than the unconjugated structure of citronellal.

The essential oil combinations of lemongrass with Shatavari and lemongrass with dill exhibited undesirable odors; hence, the mixture of lemongrass and kaffir lime leaf oils in a 1:1 ratio was selected for further investigation. Antimicrobial assays conducted against *M. furfur* identified citral—comprising geranial (trans-citral) and neral (cis-citral)—and citronellal as the primary active constituents in this essential oil mixture. As presented in Table 10, the MIC of citral was determined to be 0.03125% *v*/*v*, whereas citronellal exhibited an MIC of 0.125% *v*/*v*. Further evaluation of their combined effects revealed synergistic interactions in mixtures with ratios of 1:3 and 3:1, achieving MIC values of 0.0625% *v*/*v* and 0.03125% *v*/*v*, respectively. These findings indicated that the conjugated aldehyde structure in citral was more effective in inhibiting *M. furfur* than the unconjugated structure of citronellal.

### 2.5. Frontier Molecular Orbitals and Conjugate Additions of Essential Oils Against Malassezia furfur

The mechanisms underlying the antifungal effects of these essential oils are often attributed to the interaction of their biochemical constituents with fungal cell membranes, resulting in disruption of the cell wall and subsequent cell death. The relationship between the chemical structure of essential oils and their antifungal activity can be elucidated through frontier molecular orbital (FMO) theory. In this context, the highest occupied molecular orbital (HOMO) and the lowest unoccupied molecular orbital (LUMO) play crucial roles in determining the reactivity of these compounds [27,28]. Compounds with higher HOMO energies tend to donate electrons during reactions, whereas those with higher LUMO energies are more inclined to accept electrons. Therefore, essential oils exhibiting a favorable HOMO-LUMO gap may demonstrate increased reactivity with fungal cells, thereby enhancing their antifungal efficacy [29]. In this study, the FMO analysis in Table 11 showed that the HOMO energies of citral and citronellal were calculated to be −7.5407 eV and −6.7305 eV, respectively, indicating that citronellal has a greater propensity to donate electrons than citral. Conversely, the LUMO energies were 1.6642 eV for citral and 1.1048 eV for citronellal, suggesting that citral has a higher tendency to accept electrons. According to theoretical principles, citronellal—with a lower HOMO-LUMO energy gap of 7.8353 eV—was expected to exhibit greater reactivity than citral, which had a higher energy gap of 9.2049 eV. However, the observed antifungal activity against *M. furfur* contradicted this expectation. These results suggest that the higher LUMO energy of citral enhances its ability to accept electrons during chemical reactions, thereby promoting the disruption of fungal cell membranes and activating other pathways essential for maintaining cellular integrity.

**Figure 10:**
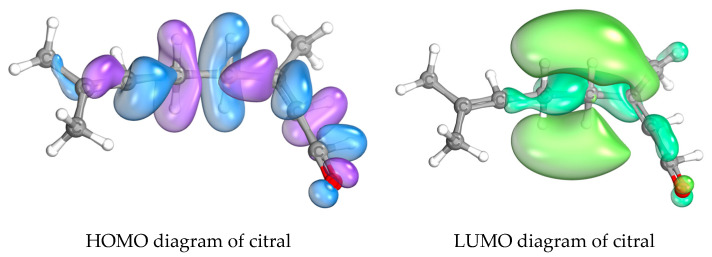

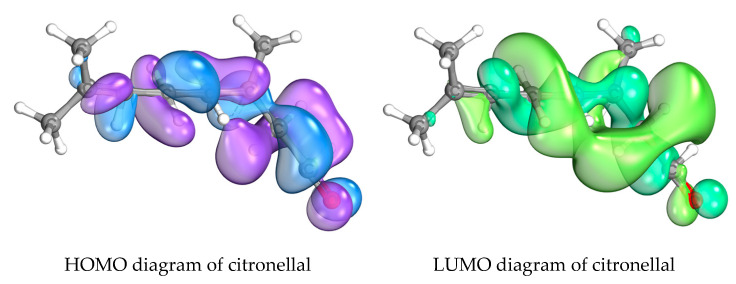
Diagrams depicting the frontier molecular orbitals of citronellal and citral, where the highest occupied molecular orbital (HOMO) is shown in violet/blue colors and the lowest unoccupied molecular orbital (LUMO) is shown in light green/dark green colors.

**Figure 11:**
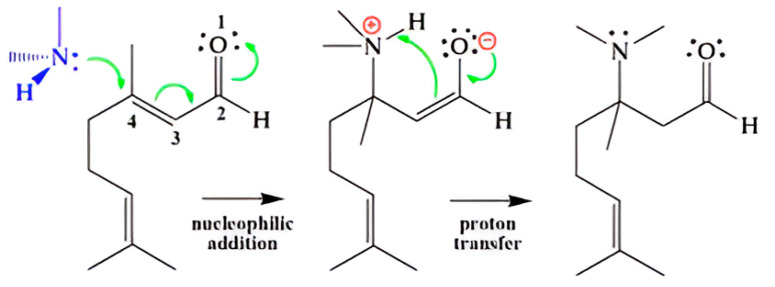
Schematic illustration of the proposed mechanism by which citral interacts with nucleophilic biomolecules within fungal cells.

The iso-density surfaces of the FMOs in Figure 10, particularly the HOMO diagrams of citral and citronellal, exhibited similar patterns; however, the LUMO diagrams showed distinct differences between these compounds. The conjugated aldehyde group in citral, along with the electron-withdrawing properties of the carbonyl group, contributed to a deficiency of electron density at carbon position 4. This conjugation considerably increased the electrophilicity of carbon position 4, rendering it more susceptible to accepting electrons from nucleophilic groups—such as amino and hydroxyl groups—present in fungal cells. This mechanism facilitated a 1,4–addition reaction, as illustrated in Figure 11. The covalent interactions between citral and biomolecules within fungal cells likely inhibited normal cellular functions, thereby contributing to its antifungal activity.

## 3. Discussion

This investigation sought to evaluate the anti-fungal potential of novel essential oil combinations derived from Lemongrass, Kaffir Lime, Lime, Dill, and Shatavari, emphasizing their chemical compositions and mechanisms of activity against *M. furfur*. The extraction yields varied markedly among the different plant sources, with dill aerial parts producing the highest essential oil quantities under hydro-distillation conditions. Such variability underscores the influence of plant material selection and extraction technique on yield efficiency, which bears significance for the scalability and commercial viability of these essential oils [30,31].

Chemical analysis via GC-MS delineated distinct profiles for each essential oil, highlighting key bioactive constituents. Lemongrass essential oil was predominantly composed of citral, which includes geranial and neral isomers; these compounds are widely acknowledged for their broad-spectrum antimicrobial and antifungal properties. [32,33]. Citral’s ability to compromise microbial cell membranes and inhibit biofilm formation provides a mechanistic basis for its efficacy. Kaffir lime essential oil primarily contained citronellal, which significantly contributes to its antimicrobial potency [34,35]. Similarly, lime essential oil was characterized by limonene, citral, and citronellal—terpenes known for their antimicrobial effects [36,37]. Dill essential oil’s main components included carvone and limonene, both exhibiting notable antifungal and antibacterial properties [38,39,40,41]. Shatavari essential oil comprises bioactive phytochemicals, such as thymol and cymene, which have been linked to antioxidant, anti-inflammatory, and antifungal effects [42,43,44].

The present study evaluated the antimicrobial activity of various essential oils and their constituents against *M. furfur*, providing valuable insights into their potential as antifungal agents. Overall, most essential oils exhibited promising antifungal activity, with lemongrass, kaffir lime, and dill essential oils demonstrating significant inhibitory effects, as evidenced by clear zones of inhibition. Notably, the combination studies revealed synergistic interactions—particularly between lemongrass and kaffir lime leaf essential oils, as well as with dill and Shatavari essential oils—suggesting that strategic blending can enhance antifungal efficacy. The observed reductions in MIC values for these combinations indicate complementary mechanisms targeting different fungal pathways, thereby increasing overall effectiveness.

Specifically, the MIC values for lemongrass and kaffir lime leaves essential oils were 0.125% *v/v* and 0.25% *v/v*, respectively. Citral, a major component of lemongrass oil, demonstrated a markedly lower MIC of 0.03125% *v/v*, consistent with its potent antifungal activity reported in previous studies [45]. Similarly, citronellal exhibited an MIC of 0.125% *v/v*, aligning with its known antimicrobial properties [21].

Synergistic effects were observed when combining lemongrass and kaffir lime essential oils in a 1:1 ratio, reducing the MIC to 0.0625% *v/v*. Additionally, the combination of citral and citronellal in a 3:1 ratio further enhanced efficacy, with an MIC of 0.03125% *v/v*. These findings highlight the potential of utilizing essential oil combinations to achieve lower effective concentrations, which may reduce toxicity risk and slow resistance development.

Comparing our results with the existing literature, Rhimi et al. (2022) reported an MIC of approximately 0.125% *v/v* (1.25 µL/mL) for lemongrass essential oil against *M. furfur*, aligning with our data [21]. Liu et al. (2022) documented an MIC of 200 µg/mL for citral, confirming its high antifungal potency [45]. Furthermore, the MICs of clove (0.422 mg/mL) and thyme (1.474 mg/mL) essential oils, along with their synergistic ratio of 1:4, reported by He et al. (2024), reinforce the effectiveness of plant-derived compounds against *M. furfur* and support the potential of combination therapies [46].

FMO analysis provided insights into the electronic interactions underpinning these bioactivities. The key constituents, citral and citronellal, exhibited differing HOMO and LUMO distributions, influencing their reactivity. Citral’s higher LUMO energy facilitated electron acceptance, aligning with increased electrophilicity, which likely enhances its capacity to disrupt fungal cell membranes. Citronellal’s lower HOMO-LUMO gap conferred greater electron-donating ability, yet its zone of activity appears to involve alternative mechanisms. Molecular orbital visualizations highlighted conjugated aldehyde groups in citral, particularly at carbon position 4, that serve as electrophilic sites, capable of engaging in nucleophilic attack within fungal cells. Such interactions may culminate in structural disruptions critical to fungal viability, corroborating the observed synergistic anti-fungal effects. In summary, this study underscores the potential of combining specific essential oils, leveraging their distinct chemical compositions and electronic properties to develop effective natural anti-fungal agents against *M. furfur*. The integration of chemical analysis and frontier molecular orbital theory offers a mechanistic understanding that can guide future formulation strategies aimed at controlling fungi implicated in dermatological conditions.

While this study provides promising in vitro anti-fungal results, several limitations necessitate further investigation. Primarily, in vivo efficacy remains unknown, requiring evaluation in animal models or clinical trials to account for bioavailability and host interactions. Additionally, although FMO analysis supports proposed mechanisms, experimental validation of essential oil-fungal cell interactions via molecular docking or biophysical methods is crucial. Cytotoxicity also remains unevaluated, underscoring the need for safety assessments on human cell lines to determine safe topical concentrations. Future research should prioritize formulation stability, skin penetration, and expanded antifungal activity against a broader range of dermatological pathogens to facilitate translational applications.

## 4. Materials and Methods

### 4.1. Plant Materials

The aerial parts of Satavari (*Asparagus racemosus* Willd.), Dill (*Anethum graveolens* L.), and lemongrass (*Cymbopogon citratus* Stapf), along with the peels of Lime (*Citrus aurantifolia* (Christm.)) and Kaffir lime (*Citrus hystrix* DC), as well as the leaves of *Citrus hystrix* DC, were collected from the Botanical Garden at the Faculty of Pharmacy, Srinakharinwirot University, Nakhon Nayok Province, Thailand, during the months of July and August. The identification of the species was confirmed by Associate Professor Sarin Tadtong, and the specimens were deposited in the herbarium of the Faculty of Pharmacy, Srinakarinwirot University, Thailand.

### 4.2. Extraction of Essential Oil

The plant materials were reduced to small pieces and subsequently extracted via hydro-distillation using a Clevenger apparatus for 2 h. The resulting essential oil was collected and stored at a temperature of 2–4 °C until its usage.

### 4.3. Essential Oil Composition Analysis

Chemical analysis was conducted using gas chromatography-mass spectrometry (GC/MS) on a Finnigan Trace GC ultra (Thermo Electron Corporation, Waltham, MA, USA), equipped with a quadrupole mass spectrometer. The column employed was a ZB-5 fused silica capillary column with a dimension of 30 m × 0.22 mm i.d. and a film thickness of 0.25 μm. The oven temperature was programmed to increase from 50 °C to 250 °C at a rate of 7 °C/min. The injector and detector temperatures were set at 250 °C and 280 °C, respectively, with a sample injection volume of 1 μL and a split ratio of 100:1. Helium was used as the carrier gas at a flow rate of 2 mL/min. Compounds were identified by comparing the Kovats gas chromatographic retention indices of the peaks on the HP-5 MS column with literature values, conducting computer matching using the MassLynx database, and analyzing the fragmentation patterns of the mass spectra in relation to those documented in the literature.

### 4.4. Inhibition Assay of Malassesia furfur Using Agar Diffusion and Broth Microdilution Methods

The inhibition of *M. furfur* was assessed utilizing the agar diffusion method and the broth microdilution method, employing modified Dixon’s medium as the growth medium. The preparation procedures are outlined as follows:

#### 4.4.1. Subculturing *Malassesia furfur*

Streak *M. furfur* onto a sterile test tube containing modified Dixon Agar. Incubate at 28 °C for 72 h.

#### 4.4.2. Preparation of Modified Dixon’s Medium

To prepare 100 mL of modified Dixon Agar, first, weigh 10.65 g of Dixon’s agar Part A and dissolve it in 100 mL of distilled water. Next, add 1.5 mL of Dixon’s Part B to the mixture and ensure thorough mixing. Autoclave the solution to achieve sterilization, and after autoclaving, allow it to cool. Finally, pour the mixture into sterile petri plates within a Class II Biohazard Safety Cabinet and permit the agar to solidify before use in experiments.

To prepare 100 mL of modified Dixon Broth, first, weigh 3.6 g of Malt Extract and 0.6 g of Peptone, place them in a container, and dissolve them in water. Then, add 2.0 g of Desiccated Oxbile, 1.0 mL of Tween 40, 0.2 mL of Glycerol, and 0.2 mL of Oleic acid to the mixture. Adjust the pH to 6 using hydrochloric acid (HCl) and subsequently autoclave the solution to ensure sterilization.

#### 4.4.3. Inhibition Assay of *Malassesia furfur* via Agar Diffusion Method

To conduct the inhibition assay of *M. furfur* via the agar diffusion method, inoculate modified Dixon Agar with *M. furfur* using a sterile swab dipped in a culture adjusted to a turbidity equivalent to McFarland Standard No. 0.5 (approximately 10^8^ CFU/mL), gently drying the swab against the side of the test tube before swabbing the agar plate surface and allowing it to stand for 3–5 min. Prepare a positive control by dissolving Ketoconazole in methanol to achieve a final concentration of 20 µg/mL. Using a micropipette, deposit 20 µL of the prepared essential oil onto sterile paper discs and place them onto the agar plate, pressing gently to adhere, with three discs per plate spaced appropriately, ensuring the experiment is repeated three times. Apply 20 µL of Ketoconazole onto a separate sterile paper disc, allowing the solvent to evaporate, and then place the Ketoconazole-coated disc onto the agar plate, also pressing gently, with one disc per plate and repeating this procedure three times as a positive control. Additionally, place a blank sterile paper disc onto the plate as a negative control, repeating this step three times. Incubate the plates at 28°C for 72 h. Subsequently, measure and record the diameter of the zones of inhibition where *M. furfur* growth is absent. Essential oils that yield inhibition zones of less than 10 mm will not be subjected to further testing.

#### 4.4.4. Inhibition Assay of *Malassesia furfur* via Broth Microdilution Method

To determine the minimum inhibitory concentration (MIC) of *M. furfur* using the broth microdilution method, first, prepare a 2% DMSO solution in modified Dixon broth. Adjust the *M. furfur* culture to a density corresponding to McFarland No. 0.5 (approximately 10^8^ CFU/mL) and dilute this suspension to a concentration of 5% in the broth. Essential oils should be prepared at varying concentrations, ensuring at least five different concentrations using 2% DMSO in the broth for two-fold dilutions. Additionally, establish a positive control by preparing Ketoconazole in 2% DMSO to achieve a final concentration of 10 µg/mL. Next, pipette the diluted microbial suspension and the various concentrations of essential oils into a 96-well plate designed for the broth microdilution method, utilizing three wells for each concentration and performing three experimental repeats. Incubate the plates at 28 °C for 72 h, and subsequently record the lowest concentration at which there is no observable growth as the MIC.

#### 4.4.5. Evaluation of the Synergistic Effects of Combined Essential Oils on *Malassesia furfur* Using the Broth Microdilution Method

The combined essential oils were prepared by mixing lemongrass oil with other essential oils in ratios of 1:1, 1:3, and 3:1. The MIC of these combinations was then determined using the method described in Section 4.4.4.

The mixed essential oils were analyzed to assess whether they exhibited synergistic, additive, or antagonistic effects by calculating the Sum of Fractional Inhibitory Concentration (ΣFIC). The interpretation of ΣFIC values is as follows:

If ΣFIC > 1, it indicates an antagonistic effect of the mixed essential oils.
If ΣFIC = 1, it signifies an additive effect of the mixed essential oils.
If ΣFIC < 1, it demonstrates a synergistic effect of the mixed essential oils.

The ΣFIC can be calculated using the formula:

$$\Sigma FIC=\frac{a}{MICA}+\frac{b}{MICB}$$

where:

MICA is the MIC of essential oil A (the first essential oil).
MICB is the MIC of essential oil B (the second essential oil).
{*a*} represents the proportion of essential oil A multiplied by the MIC of the mixed essential oils.
{*b*} represents the proportion of essential oil B multiplied by the MIC of the mixed essential oils.

### 4.5. Calculation of Frontier Molecular Orbitals and Visualization

This research employed the Avogadro software (version 1.2.0) to generate the initial chemical structures [47]. Subsequently, the optimized xyz coordinates were obtained, and Orca input (.inp) files were prepared using these coordinates, applying the restricted Hartree-Fock (RHF) method with the def2-SVP basis set. Orca [48] was then executed on these input files: an administrator command prompt was opened, the directory was changed to the location of the .inp files, and assuming Orca was added to the system PATH variable, the command used was as follows:

Orca filename.inp > filename.out

From the same job folder, a Molden file was generated using the command:

Orca_2mkl filename–molden

Finally, the Molden files were visualized with IboView software (version 20211019) [49], which was employed for intrinsic bond orbital (IBO) calculations, as well as for analyzing geometric structures and molecular orbital (MO) representations.

## 5. Conclusions

This study underscores the potential of essential oil combinations as effective anti-fungal agents against *M. furfur*. The integration of chemical characterization with biological activity assessments provided a comprehensive understanding of how specific constituents contribute to antifungal efficacy. Among the oils tested, lemongrass demonstrated notable activity, with an MIC of 0.125% *v*/*v*, while citral—the primary active compound in lemongrass—exhibited a more potent MIC of 0.03125% *v*/*v*. Synergistic effects were observed when lemongrass oil was combined with kaffir lime, dill, or Shatavari essential oils in a 1:1 ratio, enhancing anti-fungal activity against *M. furfur*. Additionally, citral demonstrated synergy with citronellal at ratios of 1:3 and 3:1, further amplifying anti-fungal effects. The antifungal mechanism of citral is attributed to its electron acceptor properties and its facilitation of 1,4-nucleophilic addition, leading to covalent bond formation with nucleophilic molecules in fungal cells, ultimately resulting in fungal cell death. Future research should focus on optimizing these combinations to improve stability, efficacy, and sensory qualities, thereby advancing their application in anti-dandruff formulations. Furthermore, elucidating the precise molecular mechanisms underlying the antifungal activity of these essential oils could provide deeper insights into their mode of action and foster the development of innovative therapeutic strategies.

## 6. Patents

The antifungal essential oil formulation against *Malassezia furfur* consists of lemongrass oil and kaffir lime leaf oil in a 1:1 ratio and was reported in this manuscript.

## Figures and Tables

**Table 1:** Hydro-distillation extraction yields of essential oils from lemongrass leaves, kaffir lime peels, kaffir lime leaves, lime peels, dill aerial parts, and Shatavari aerial parts (*n* = 3).

Plant Samples	Dill Aerial Part	Kaffir Lime Peels	Lime Peels	Shatavari Areal Part	Lemongrass	Kaffir Lime Leaves
Yield (%*w*/*w*)	1.08 ± 0.11	0.86 ± 0.12	0.68 ± 0.08	0.65 ± 0.09	0.61 ± 0.08	0.27 ± 0.05

**Table 2:** Chemical composition of essential oils from lemongrass aerial parts.

RT	Name Compound	Chemical Class	Kovats Index	Area%
19.82	Geranial	Oxygenated Monoterpenes	1267	45.03
18.41	Neral	Oxygenated Monoterpenes	1238	27.07
18.82	Geraniol	Oxygenated Monoterpenes	1252	4.52
22.3	Citral<dimethoxy-(E)->	Oxygenated Monoterpenes	1341	3.50
34.07	Selina-6-en-4-ol	Oxygenated Sesquiterpenes	1624	3.38
24.19	Geranyl acetate	Oxygenated Monoterpenes	1381	3.28
23.67	Neric acid	Oxygenated Monoterpenes	1368	2.44
21.27	Citral<dimethoxy-(Z)->	Oxygenated Monoterpenes	1318	1.75
15.72	Isocitral<E->	Oxygenated Monoterpenes	1180	1.49
17.73	Citronellol	Oxygenated Monoterpenes	1225	1.16
51.28	Unidentified	Other		1.71
14.89	Isocitral<Z->	Oxygenated Monoterpenes	1164	0.85
7.99	Myrcene	Monoterpene Hydrocarbons	990	0.83
35.25	Cadinol<alpha->	Oxygenated Sesquiterpenes	1654	0.74
12.26	Linalool	Oxygenated Monoterpenes	1096	0.71
56.85	Unidentified	Other		0.64
35.6	Juniper camphor	Oxygenated Monoterpenes	1663	0.61
34.79	Murrolol<epi-alpha-> (=tau-muurolol)	Oxygenated Sesquiterpenes	1642	0.39
36.82	Eudesm-7(11)-en-4-ol	Oxygenated Sesquiterpenes	1700	0.25
33.4	Eudesmol<5-epi-7-epi-alpha->	Oxygenated Sesquiterpenes	1607	0.16
32.43	Caryophyllene oxide	Oxygenated Sesquiterpenes	1583	0.14
	Sums of percentage of oxygenated monoterpenes			92.41
	Sums of percentage oxygenated sesquiterpenes			5.06
	Sums of percentage of monoterpene hydrocarbons			0.83
	Sums of percentage of other compounds			2.35
	Total percentage of all identified compounds			97.65

**Table 3:** Chemical composition of essential oils from kaffir lime leaves.

RT	Name Compound	Chemical Class	Kovats Index	Area%
14.74	Citronellal	Oxygenated Monoterpenes	1153	83.76
17.75	Citronellol	Oxygenated Monoterpenes	1225	5.20
22.99	Citronellyl acetate	Oxygenated Monoterpenes	1352	4.61
12.26	Linalool	Oxygenated Monoterpenes	1096	2.04
7.54	Sabinene	Monoterpene Hydrocarbons	975	0.85
31.58	Nerolidol<E->	Oxygenated Sesquiterpenes	1563	0.55
11.14	Linalool oxide <cis-> (furanoid)	Oxygenated Monoterpenes	1072	0.52
7.99	Myrcene	Monoterpene Hydrocarbons	990	0.51
24.21	Geranyl acetate	Oxygenated Monoterpenes	1381	0.42
11.78	Linalool oxide<trans-> (furanoid)	Oxygenated Monoterpenes	1086	0.3
31.11	Elemol	Oxygenated Sesquiterpenes	1549	0.25
25.83	Caryophyllene(E-)	Oxygenated Sesquiterpenes	1419	0.24
15.78	Terpinen-4-ol	Oxygenated Monoterpenes	1177	0.22
10.06	Ocimene<(E)-beta->	Monoterpene Hydrocarbons	1050	0.21
29.82	Amorphene<delta->	Sesquiterpene Hydrocarbons	1512	0.14
28.95	Bicyclogermacrene	Sesquiterpene Hydrocarbons	1500	0.13
27.32	Humulene<alpha->	Sesquiterpene Hydrocarbons	1454	0.04
	Sums of percentage of oxygenated monoterpenes			97.62
	Sums of percentage oxygenated sesquiterpenes			1.04
	Sums of percentage of monoterpene hydrocarbons			1.57
	Sums of percentage of sesquiterpene hydrocarbons			0.31
	Sums of percentage of other compounds			0.00
	Total percentage of all identified compounds			100.00

**Table 4:** Chemical composition of essential oils from kaffir lime peels.

RT	Name Compound	Chemical Class	Kovats Index	Area%
7.72	Pinene<beta->	Monoterpene Hydrocarbons	979	29.49
9.5	Sylvestrene	Monoterpene Hydrocarbons	1030	20.77
14.56	Citronellal	Oxygenated Monoterpenes	1153	14.02
7.58	Sabinene	Monoterpene Hydrocarbons	975	7.49
17.75	Citronellol	Oxygenated Monoterpenes	1225	5.80
15.78	Terpinen-4-ol	Oxygenated Monoterpenes	1177	5.27
16.44	Terpineol<alpha->	Oxygenated Monoterpenes	1188	4.48
12.26	Linalool	Oxygenated Monoterpenes	1096	2.05
6.36	Pinene<alpha->	Monoterpene Hydrocarbons	939	1.83
11.14	Linalool oxide <cis-> (furanoid)	Oxygenated Monoterpenes	1072	1.80
11.76	Linalool oxide<trans-> (furanoid)	Oxygenated Monoterpenes	1086	1.07
10.56	Terpinene<gamma->	Monoterpene Hydrocarbons	1059	1.04
29.84	Cadinene<delta->	Sesquiterpene Hydrocarbons	1523	0.89
23.96	Copaene<alpha->	Sesquiterpene Hydrocarbons	1376	0.64
24.5	Cubebene<beta->	Sesquiterpene Hydrocarbons	1388	0.46
18.74	Geraniol	Oxygenated Monoterpenes	1252	0.40
22.97	Citronellyl acetate	Oxygenated Monoterpenes	1352	0.40
25.83	Caryophyllene(E-)	Sesquiterpene Hydrocarbons	1419	0.32
13.38	Menth-2-en-1ol<trans-para->	Oxygenated Monoterpenes	1140	0.30
24.21	Geranyl acetate	Oxygenated Monoterpenes	1381	0.23
28.35	Germacrene D	Sesquiterpene Hydrocarbons	1485	0.23
29.04	Muurolene<alpha->	Sesquiterpene Hydrocarbons	1500	0.22
31.11	Elemol	Oxygenated Sesquiterpenes	1549	0.21
27.32	Humulene<alpha->	Sesquiterpene Hydrocarbons	1454	0.18
47.78	Unidentified	Other		0.16
35.23	Cadinol<alpha->	Oxygenated Sesquiterpenes	1654	0.13
22.72	Menthol<8-hydroxy-neo->	Oxygenated Monoterpenes	1330	0.12
	Sums of percentage of monoterpene hydrocarbons			60.62
	Sums of percentage of oxygenated monoterpenes			35.94
	Sums of percentage oxygenated sesquiterpenes			0.34
	Sums of percentage of sesquiterpene hydrocarbons			2.62
	Sums of percentage of other compounds			0.16
	Total percentage of all identified compounds			99.84

**Table 5:** Chemical composition of essential oils from kaffir lime peels.

RT	Name Compound	Chemical Class	Kovats Index	Area%
9.55	Sylvestrene	Monoterpene Hydrocarbons	1030	62.29
7.72	Pinene<beta->	Monoterpene Hydrocarbons	979	15.33
10.58	Terpinene<gamma->	Monoterpene Hydrocarbons	1059	3.48
6.34	Pinene<alpha->	Monoterpene Hydrocarbons	939	2.82
29.45	Bisabolene<beta->	Sesquiterpene Hydrocarbons	1505	2.31
23.36	Neryl acetate	Oxygenated Monoterpenes	1361	1.91
19.57	Geranial	Oxygenated Monoterpenes	1267	1.82
26.36	Bergamotene<alpha-trans->	Sesquiterpene Hydrocarbons	1434	1.55
16.42	Terpineol<alpha->	Oxygenated Monoterpenes	1188	1.53
18.26	Neral	Oxygenated Monoterpenes	1238	1.52
17.62	Nerol	Oxygenated Monoterpenes	1229	1.31
18.74	Geraniol	Oxygenated Monoterpenes	1252	0.60
15.74	Terpinen-4-ol	Oxygenated Monoterpenes	1177	0.57
12.26	Linalool	Oxygenated Monoterpenes	1096	0.54
32.43	Caryophyllene oxide	Oxygenated Sesquiterpenes	1583	0.48
21.23	Citral<dimethoxy-(Z)->	Oxygenated Monoterpenes	1318	0.45
22.22	Citral<dimethoxy-(E)->	Oxygenated Monoterpenes	1341	0.29
25.82	Caryophyllene(E-)	Sesquiterpene Hydrocarbons	1419	0.27
24.21	Geranyl acetate	Oxygenated Monoterpenes	1381	0.26
25.56	Bergamotene<alpha-cis->	Sesquiterpene Hydrocarbons	1412	0.17
36.41	Bisabolol<alpha->	Oxygenated Sesquiterpenes	1685	0.17
35.39	Unidentified	Other		0.14
35.87	Unidentified	Other		0.13
34.73	Unidentified	Other		0.06
	Sums of percentage of monoterpene hydrocarbons			83.92
	Sums of percentage of oxygenated monoterpenes			11.45
	Sums of percentage oxygenated sesquiterpenes			0.65
	Sums of percentage of sesquiterpene hydrocarbons			4.30
	Sums of percentage of other compounds			0.33
	Total percentage of all identified compounds			99.67

**Table 6:** Chemical composition of essential oils from dill aerial parts.

RT	Name Compound	Chemical Class	Kovat’s Index	Area%
8.7	Phellandrene<alpha->	Monoterpene Hydrocarbons	1002	43.54
16.09	Dill ether	Oxygenated Monoterpenes	1186	25.24
9.55	Phellandrene<beta->	Monoterpene Hydrocarbons	1029	10.46
9.42	Cymene<ortho->	Monoterpene Hydrocarbons	1026	5.05
18.8	Limonene dioxide	Monoterpene Hydrocarbons	1251	3.45
30.15	Myristicin	Other	1518	3.26
22.18	Unidentified	Other		2.91
6.34	Pinene<alpha->	Monoterpene Hydrocarbons	939	1.99
26.11	Barosma camphor	Oxygenated Monoterpenes	1427	0.92
25.06	2,3-Bornanediol	Other	1410	0.87
21.93	Pinanediol<cis-2,3->	Oxygenated Monoterpenes	1320	0.48
26.47	2-Cyclohexen-1-one, 4-hydroxy-3-methyl-6-(1-methylethyl)-, trans-	Oxygenated Monoterpenes	1436	0.47
8.01	Myrcene	Monoterpene Hydrocarbons	990	0.46
20.96	Unidentified	Other		0.44
20.61	Unidentified	Other		0.36
7.54	Sabinene	Monoterpene Hydrocarbons	975	0.11
	Sums of percentage of monoterpene hydrocarbons			65.06
	Sums of percentage of oxygenated monoterpenes			27.11
	Sums of percentage of other compounds			7.84
	Total percentage of all identified compounds			96.29

**Table 7:** Chemical composition of essential oils from Shatavari aerial parts.

RT	Name Compound	Chemical Class	Kovats Index	Area%
8.7	Phellandrene<alpha->	Monoterpene Hydrocarbons	1002	26.14
17.83	Thymol, methyl ether	Oxygenated Monoterpenes	1235	18.07
9.36	Cymene<ortho->	Monoterpene Hydrocarbons	1026	10.30
28.33	Germacrene D	Sesquiterpene Hydrocarbons	1485	10.03
25.66	Cymene<2,5-dimethoxy-para->	Oxygenated Monoterpenes	1426	5.17
7.72	Pinene<beta->	Monoterpene Hydrocarbons	979	4.00
7.97	Myrcene	Monoterpene Hydrocarbons	990	2.90
18.8	Limonene dioxide	Oxygenated Monoterpenes	1251	2.70
31.83	Unidentified	Other		2.08
33.16	Unidentified	Other		1.62
22.18	Unidentified	Other		1.60
22.32	Unidentified	Other		1.56
25.1	2,3-Bornanediol	Other	1401	1.43
21.95	Pinanediol<cis-2,3->	Oxygenated Monoterpenes	1320	1.42
10.06	Ocimene<(E)-beta->	Monoterpene Hydrocarbons	1050	1.14
6.34	Pinene<alpha->	Monoterpene Hydrocarbons	939	1.07
26.51	Unidentified	Other		1.05
26.16	Piperitone oxide	Oxygenated Monoterpenes	1428	0.96
38.89	Unidentified	Other		0.79
18.43	Mesityl methyl ketone	Other	1239	0.77
42.13	Unidentified	Other		0.72
27.32	Humulene<alpha->	Sesquiterpene Hydrocarbons	1454	0.67
35.23	Cadinol<alpha->	Oxygenated Sesquiterpenes	1654	0.51
20.96	Unidentified	Other		0.47
20.58	Unidentified	Other		0.45
20.19	Bornyl acetate	Oxygenated Monoterpenes	1288	0.43
43.62	Unidentified	Other		0.43
34.38	Unidentified	Other		0.42
39.91	Unidentified	Other		0.42
16.75	Pinocarveol<cis->	Oxygenated Monoterpenes	1184	0.35
34.73	Cadinol<epi-alpha-> (=tau-cadinol)	Oxygenated Sesquiterpenes	1640	0.31
	Sums of percentage of monoterpene hydrocarbons			45.55
	Sums of percentage of oxygenated monoterpenes			29.10
	Sums of percentage of sesquiterpene hydrocarbons			10.70
	Sums of percentage oxygenated sesquiterpenes			0.82
	Sums of percentage of other compounds			13.81
	Total percentage of all identified compounds			88.39

**Table 8:** Diameters of inhibition zones and MIC values of essential oils from lemongrass, kaffir lime peels, kaffir lime leaves, lime peels, dill aerial parts, and Shatavari aerial parts (n = 3).

Plant Samples	Lemongrass	Kaffir Lime Leaves	Kaffir Lime Peels	Lime Peels	Dill Aerial Parts	Shatavari Aerial Parts
Diameter (cm ± SD)	NMGO	NMGO	NMGO	NMGO	NMGO	1.05 ± 0.12
MIC (% *v*/*v*)	0.125	0.25	0.50	1.00	0.25	0.25

NMGO = no microbial growth observed.

**Table 9:** Synergistic effects of combined essential oils on *Malassezia furfur*.

Combined Essential Oils	Ratios	MIC (% *v*/*v*)	ΣFIC	Interpretation
Lemongrass and Lime peels essential oils	1:3	0.25	2.75	Antagonism
	1:1	0.125	1.125	Antagonism
	3:1	0.125	3.125	Antagonism
Lemongrass and Kaffir lime leaves essential oils	1:3	0.25	3.5	Antagonism
	1:1	0.125	1.25	Antagonism
	3:1	0.0625	1.625	Antagonism
Lemongrass and Kaffir lime peels essential oils	1:3	0.0625	1.25	Antagonism
	1:1	0.0625	0.75	Synergism
	3:1	0.0625	1.75	Antagonism
Lemongrass and Dill essential oils	1:3	0.0625	1.25	Antagonism
	1:1	0.0625	0.75	Synergism
	3:1	0.0625	1.75	Antagonism
Lemongrass and Shatavari essential oils	1:3	0.0625	1.25	Antagonism
	1:1	0.0625	0.75	Synergism
	3:1	0.0625	1.75	Antagonism

**Table 10:** Synergistic effects of citral and citronellal against *Malassezia furfur*.

Combined Essential Oils	Ratios	MIC (% *v*/*v*)	ΣFIC	Interpretation
Citral	–	0.03125	–	–
Citronellal	–	0.125	–	–
Citral and Citronellal	1:3	0.0625	0.875	Synergism
	1:1	0.0625	1.25	Antagonism
	3:1	0.03125	0.8125	Synergism

**Table 11:** Calculated highest occupied molecular orbital (HOMO) and the lowest unoccupied molecular orbital (LUMO) energies of citral and citronellal in electron volts (eV).

Essential Oils	HOMO (eV)	LUMO (eV)	Energy Gap (eV)
Citral	−7.5407	1.6642	9.2049
Citronellal	−6.7305	1.1048	7.8353

## Data Availability

Data are contained within the article.

## Abbreviations

The following abbreviations are used in this manuscript:

CFU	Colony-forming unit
DMSO	Dimethyl sulfoxide
FMO	Frontier molecular orbital
GC-MS	Gas chromatography-mass spectrometry
HCl	Hydrochloric acid
HOMO	Highest occupied molecular orbital
IBO	Intrinsic bond orbital
LUMO	Lowest unoccupied molecular orbital
MIC	Minimum inhibitory concentration
MO	Molecular orbital
mL	Milliliter
NMGO	no microbial growth observed
i.d.	Internal diameter
ΣFIC	Sum of fractional inhibitory concentrations
°C	Degree Celsius
µg	Microgram
µL	Microliter
µm	Micrometer
